# Vaccination prior to SARS-CoV-2 infection does not affect the neurologic manifestations of long COVID

**DOI:** 10.1093/braincomms/fcae448

**Published:** 2025-01-07

**Authors:** Shreya Mukherjee, Tracey Singer, Aditi Venkatesh, Natasha A Choudhury, Gina S Perez Giraldo, Millenia Jimenez, Janet Miller, Melissa Lopez, Barbara A Hanson, Aasheeta P Bawa, Ayush Batra, Eric M Liotta, Igor J Koralnik

**Affiliations:** Davee Department of Neurology, Northwestern University Feinberg School of Medicine, Chicago, IL 60611, USA; Davee Department of Neurology, Northwestern University Feinberg School of Medicine, Chicago, IL 60611, USA; Davee Department of Neurology, Northwestern University Feinberg School of Medicine, Chicago, IL 60611, USA; Davee Department of Neurology, Northwestern University Feinberg School of Medicine, Chicago, IL 60611, USA; Davee Department of Neurology, Northwestern University Feinberg School of Medicine, Chicago, IL 60611, USA; Davee Department of Neurology, Northwestern University Feinberg School of Medicine, Chicago, IL 60611, USA; Northwestern Medicine, Chicago, IL 60611, USA; Davee Department of Neurology, Northwestern University Feinberg School of Medicine, Chicago, IL 60611, USA; Davee Department of Neurology, Northwestern University Feinberg School of Medicine, Chicago, IL 60611, USA; Northwestern Medicine, Chicago, IL 60611, USA; Davee Department of Neurology, Northwestern University Feinberg School of Medicine, Chicago, IL 60611, USA; Davee Department of Neurology, Northwestern University Feinberg School of Medicine, Chicago, IL 60611, USA; Davee Department of Neurology, Northwestern University Feinberg School of Medicine, Chicago, IL 60611, USA

**Keywords:** COVID-19, SARS-CoV-2 infection, long COVID, post-acute sequelae of SARS-CoV-2 infection, neurology

## Abstract

Persistent symptoms after COVID-19 constitute the long COVID syndrome, also called post-acute sequelae of SARS-CoV-2 infection (PASC). COVID-19 vaccines reduce the gravity of ensuing SARS-CoV-2 infections. However, whether vaccines also have an impact on PASC remain unknown. We investigated whether vaccination prior to infection alters the subsequent neurologic post-acute sequelae of SARS-CoV-2 infection (Neuro-PASC). We studied prospectively the first consecutive 200 post-hospitalization Neuro-PASC (PNP) and 1100 non-hospitalized Neuro-PASC (NNP) patients evaluated at our neuro-COVID-19 clinic between May 2020 and January 2023. Among PNP patients, 87% had a pre-vaccination infection and 13% had a breakthrough infection post-vaccination. Among the NNP patients, 70.7% had a pre-vaccination infection and 29.3% had a breakthrough infection. Both PNP and NNP breakthrough infection patients had more frequent pre-existing depression/anxiety than their respective pre-vaccination infection groups, and NNP breakthrough infection patients also had more frequent comorbidities of headache, lung and gastrointestinal diseases than the NNP pre-vaccination infection group. An average of 10 months after symptom onset, the three most common neurological symptoms for PNP patients were brain fog (86.5%), numbness/tingling (56.5%) and headache (56.5%). Of all Neuro-PASC symptoms, PNP breakthrough infection more frequently reported anosmia compared to PNP pre-vaccination infection patients (69.2 versus 37.9%; *P* = 0.005). For NNP patients, the three most common neurological symptoms were brain fog (83.9%), headache (70.9%) and dizziness (53.8%). NNP pre-vaccination infection reported anosmia (56.6 versus 39.1%; *P* < 0.0001) and dysgeusia (53.3 versus 37.3%; *P* < 0.0001) more frequently than breakthrough infection patients. NNP breakthrough infection more frequently reported dizziness compared to NNP pre-vaccination infection patients (61.5 versus 50.6%; *P* = 0.001). Both PNP and NNP patients had impaired quality-of-life in cognitive, fatigue, sleep, anxiety and depression domains with no differences between pre-vaccination infection and breakthrough infection groups. PNP patients performed worse on National Institutes of Health Toolbox tests of processing speed, attention, executive function and working memory than a US normative population whereas NNP patients had lower results in processing, speed, attention and working memory, without differences between pre-vaccination infection and breakthrough infection groups. These results indicate that vaccination prior to SARS-CoV-2 infection does not affect the neurologic manifestations of long COVID in either PNP or NNP patients. Minor differences in neurologic symptoms between pre-vaccination infection and breakthrough infection groups may be caused by SARS-CoV-2 strains evolution. Patients developing Neuro-PASC after breakthrough infection have a higher burden of comorbidities, highlighting different risk factors warranting targeted management.

## Teaser

Mukherjee *et al*. report that vaccination prior to SARS-CoV-2 infection does not affect the neurological manifestations of Long COVID. Minor symptom differences may result from viral strain evolution. Patients with breakthrough infections have a higher burden of comorbidities, highlighting the need for targeted management based on distinct risk factors.

## Graphical Abstract

**Graphical Abstract:**
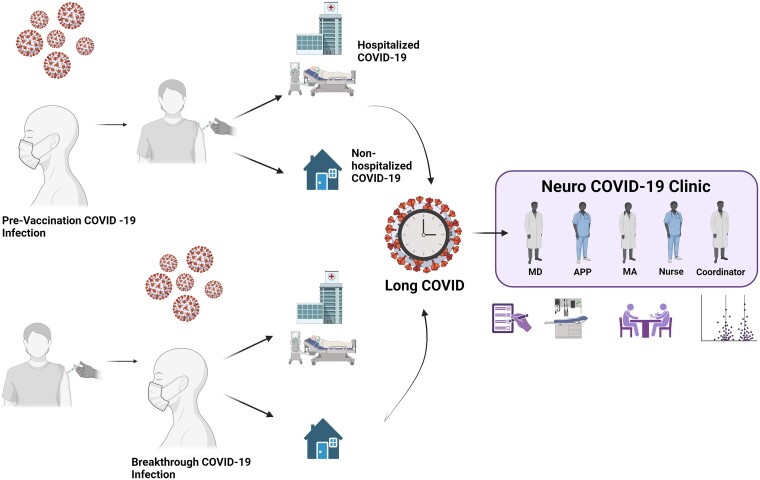

## Introduction

As of October 2024, there have been more than 776 million reported cases of COVID-19 globally and over 7 million deaths. In the United States alone, there have been over 103 million cases and 1.2 million deaths from COVID-19.^1^ Although COVID-19 is primarily a respiratory disease, it also affects multiple organs in the body including the nervous system. Furthermore, while many people only develop transient and mild initial respiratory symptoms of COVID-19, some develop lingering respiratory, cardiovascular, gastrointestinal and neurologic symptoms that constitute the ‘long COVID’ syndrome or ‘post-acute sequelae of SARS-CoV-2 infection’ (PASC). A household pulse survey carried out by the National Center for Health Statistics estimates that as of 24 June 2024, 18.4% of all US adults ever experienced long COVID.^2^

We and others have shown differences in the incidence and characteristics of PASC in patients who had previously been hospitalized with COVID-19 pneumonia compared to non-hospitalized individuals who had a mild initial respiratory presentation.^3-6^ Particularly, we recently characterized differences in neurological signs and symptoms, and cognitive dysfunction that post-hospitalization Neuro-PASC (PNP) and non-hospitalized Neuro-PASC (NNP) patients experience.^5^ While vaccination status was included as a demographic characteristic of the patients, a detailed analysis of the independent effect of COVID-19 vaccination on the severity of neurological symptoms in PNP and NNP patients is needed.

COVID-19 vaccines only prevent an estimated 52% of expected infections.^7^ Prior studies have investigated the incidence of PASC in people who were infected with SARS-CoV-2 before and after vaccination. Systematic reviews of large COVID cohorts across various countries show a modest protective effect of vaccination from the subsequent development of long COVID.^8-11^ An electronic health record-based cohort study in the US showed that, compared to unvaccinated individuals, those with complete vaccine series prior to infection had a 30% decrease in the odds of long COVID clinical diagnosis.^12^ In a large cohort of patients from the US Department of Veteran Affairs database, breakthrough infection (BTI) patients showed a 15% risk reduction of long COVID compared to those who were infected but were not previously vaccinated.^13^ Other studies have shown that individuals who received a COVID-19 vaccine at least 2 weeks before SARS-CoV-2-infection had a reduced risk of some but not all sequelae 6 months after infection when compared with individuals unvaccinated for COVID-19 but who had received an influenza vaccine, although it did not alter long COVID features.^14^

Conversely, whether vaccination has an impact on established long COVID symptoms remains a matter of debate. A recent meta-analysis was inconclusive, with some studies showing improvement while others showed no change or worsening of long COVID symptoms after vaccination.^9,15^ Given that symptoms of long COVID can lead to significant lasting impairment to patients, it is necessary to understand how to protect against the development of this condition. However, there has yet to be a study showing a detailed characterization of neurologic symptoms and manifestations, as well as quality-of-life and cognitive function in PNP and NNP patients who were infected before or after vaccination.

Since COVID-19 vaccines reduce the gravity of following SARS-CoV-2 infections,^16^ we hypothesized that they may also affect the neurologic manifestations of subsequent long COVID. Therefore, we sought to prospectively evaluate the neurologic symptoms, cognitive dysfunction and quality-of-life in PNP and NNP patients, with respect to those who experienced pre-vaccination infection (PVI) defined as having a SARS-CoV-2 infection before any SARS-CoV-2 vaccination, or a BTI, defined as SARS-CoV-2 infection more than 2 weeks after receiving any SARS-CoV-2 vaccination. We aimed to identify what effects, if any, vaccination prior to COVID-19 infection has on Neuro-PASC manifestations as well as quality-of-life and cognitive function in PNP and NNP patients.

## Materials and methods

### Patients

We evaluated the first 200 consecutive PNP and 1100 NNP patients who were SARS-CoV-2-positive at the Neuro-COVID-19 clinic of Northwestern Memorial Hospital, in Chicago, Illinois, between its opening in May 2020 and January 2023. The clinic was listed on a webpage without further advertising.^17^ Patients could schedule appointments in-person or through televisits without need for physician referral, as previously noted.^5^

Inclusion criteria for this study were the same as previously published.^5^ Briefly, all patients must have had COVID-19, confirmed by positive SARS-CoV-2 PCR or positive rapid antigen testing, and/or positive serology for SARS-CoV-2 prior to vaccination. Additionally, patients must have had at least 6 weeks of persisting neurologic symptoms since confirmed COVID-19 infection. This definition is more stringent than that of the centers for disease control (CDC) (>4 weeks of symptoms).^18^ The patients included in this study also fit both the WHO long COVID criteria and the National Institutes of Health (NIH) PASC criteria.^19,20^ This study was approved by Northwestern University IRB (STU00212583). Of note, the first 600 patients evaluated in the clinic have been reported previously to compare neurologic manifestations between two groups of 100 PNP and 500 NNP patients.^5^ None of those inter-group analyses were reduplicated in the present study, which focuses solely on whether SARS-CoV-2 vaccination prior to infection affects the severity of long COVID within each group of PNP and NNP patients.

### Procedures

Evaluation of all patients was performed in-person or in video-conference televisit as previously published.^5^ Briefly, televisits allowed the inclusion of patients from across 37 US states. Each appointment lasted 1 h using a standardized template for all patients. Patients answered validated Patient Reported Outcome Measurement Information System (PROMIS) questionnaires about their subjective quality-of-life prior to the office visit.^21,22^ Lastly, patients were asked their subjective % of recovery compared their pre-COVID baseline.

Patients seen in-person and those evaluated initially in televisit who could come to our clinic within a week of their appointment completed an objective evaluation of their cognition with the NIH Toolbox v2.1 as previously published.^5,23-26^

### Statistical analysis

Demographics, comorbidities and signs and symptoms, were summarized as either frequency (with percentage), mean (with SD) of patients for normally distributed variables or median [with inter-quartile range (IQR)] for non-normally distributed variables. Differences in demographics were assessed using Mann–Whitney and Fisher’s exact test. Comparisons between comorbidities of the groups used χ^2^ analysis or Fisher’s exact test for groups with <5 patients. Mann–Whitney test was performed to evaluate months from onset, per cent recovery and number of symptoms present for the signs and symptoms data. Group differences for a specific sign or symptom were assessed using χ^2^ analysis or Fisher’s exact test for groups with <5 patients. Between group PROMIS and Toolbox results were summarized as a median with IQR. Mann–Whitney test was performed for comparisons within PROMIS and Toolbox scores. Two-sided *P* ≤ 0.05 was considered significant and analyses were performed in GraphPad Prism version 9.0.0. All data from the study were collected and organized in a REDCap database.

To summarize and visualize the multidimensional symptom profiles of the PNP and NNP cohorts and the relationships between PASC symptoms, we performed multiple correspondence analyses (MCA) for the PNP and NNP cohorts using those symptoms reported as present in 20% or more of patients. MCA results are presented graphically as patient and symptom point clouds in 2D space defined by the first and second principal component dimensions (the two orthogonal axes with the largest portion of the data inertia or amount of variation explained by the component). In the MCA graphs, points further from the origin have greater influence on the component axes, patients plotted in similar locations in space have similar symptom profiles, and symptom categories with similar profiles of patients are grouped together. Patients were colour-coded by vaccination status with concentration ellipses added to the graphs by vaccination group. MCA was performed using the FactoMineR package in R (R version 4.2.1, Vienna, Austria).

## Results

### Patient demographics and comorbidities

Of the 200 PNP included in this study, the mean age was 55.6 years, 55.5% were female, 60.5% were White, 20.5% Black, 3.5% Asian and 16.5% were Hispanic. Among the PNP, 87% of patients were classified as having a ‘PVI’, defined as having a COVID-19 infection before any SARS-CoV-2 vaccination, while 13% of the patients were classified as having a ‘BTI’, defined as an infection more than 2 weeks after receiving any SARS-CoV-2 vaccination. Of the 1100 NNP patients, the mean age was 46.2 years, 67.1% were female, 74.8% were White, 7.9% Black, 3.8% Asian and 10.8% were Hispanic. In the NNP group, 70.7% had PVI and 29.3% had BTI. NNP patients with BTI more frequently had in-person visits than NNP patients with PVI (66.8 versus 48.6%; *P* < 0.0001). While 100% of PNP and NNP patients with BTI had been vaccinated, 79.3% of PNP PVI and 78.4% of NNP PVI patients had received the COVID-19 vaccine by the time of their clinic visit. Demographics are shown in Table 1.

**Table 1:** Demographics for PVI and BTI in PNP and NNP patients

	Overall PNP	PNP PVI	PNP BTI	*P*	Overall NNP	NNP PVI	NNP BTI	*P*
*n* (%)	200	174 (87)	26 (13)	**<0**.**0001**	1100	778 (70.7)	322 (29.3)	**<0**.**0001**
Age, years [mean (1 SD)]	55.6(14)	55.2 (14.2)	55 (13)	0.7	46.2 (14)	45.8 (13.9)	47.3 (14.4)	0.2
Gender, *n* (%)				0.51				0.1
Male, *n* (%)	89 (44.5)	79 (45.4)	10 (38.5)		362 (32.9)	267 (34.3)	95 (29.5)	
Female, *n* (%)	111 (55.5)	95 (54.6)	16 (61.5)		738 (67.1)	511 (65.7)	227 (70.5)	
Race, *n* (%)				0.73				0.2
White	121 (60.5)	107 (61.5)	14 (53.8)		823 (74.8)	592 (76.1)	231 (71.7)	
Black or African American	41 (20.5)	34 (19.5)	7 (26.9)		87 (7.9)	60 (7.7)	27 (8.4)	
Asian	7 (3.5)	5 (2.8)	2 (7.7)		42 (3.8)	22 (2.8)	20 (6.2)	
American Indian/Alaskan Native	3 (1.5)	3 (1.7)	0 (0)		3 (0.3)	2 (0.3)	1 (0.3)	
Native Hawaiian/Pacific Islander	1 (0.5)	1 (0.57)	0 (0)		2 (0.2)	1 (0.1)	1 (0.3)	
Other	16 (8)	14 (8)	2 (7.7)		73 (6.6)	54 (6.9)	19 (5.9)	
Multiracial	5 (2.5)	5 (2.9)	0 (0)		13 (1.2)	10 (1.3)	3 (0.9)	
Not specified	6 (3)	5 (2.9)	1 (3.8)		57 (5.2)	37 (4.8)	20 (6.2)	
Ethnicity, *n* (%)				1				0.9
Not hispanic or Latino	161 (80.5)	141 (81)	20 (76.9)		920 (83.6)	651 (83.7)	269 (83.5)	
Hispanic or Latino	33 (16.5)	28 (16.1)	5 (19.2)		119 (10.8)	85 (10.9)	34 (10.6)	
Not specified	6 (3)	5 (2.9)	1 (3.8)		61 (5.5)	42 (5.4)	19 (5.9)	
Visit type, *n* (%)				0.19				**<0**.**0001**
In-person	115 (57.5)	97 (55.7)	18 (69.2)		593 (53.9)	378 (48.6)	215 (66.8)	
Televisit	85 (42.5)	77 (44.3)	8 (30.8)		507 (46.1)	400 (51.4)	107 (33.2)	
SARS-CoV-2 vaccination status at time of visit, *n* (%)				**0**.**03**				**<0**.**0001**
Yes	164 (82)	138 (79.3)	26 (100)		932 (84.7)	610 (78.4)	322 (100)	
No	26 (13)	26 (14.9)	0 (0)		125 (11.4)	125 (16.1)	0 (0)	
Unknown	10 (5)	10 (5.7)	0 (0)		43 (3.9)	43 (5.5)	0 (0)	

*P*-values that are significant *P* < 0.05 are highlighted in bold.

The distribution of comorbidities varied between the two vaccination groups for both PNP and NNP patients. PNP BTI patients were more likely than PNP PVI patients to have depression/anxiety prior to COVID-19 (34.6 versus 14.4%; *P* = 0.02). NNP BTI compared to PVI patients had more frequent depression/anxiety (29.5 versus 21.9%; *P* = 0.01), lung diseases (22 versus 15%; *P* = 0.006), headache (17.4 versus 12.1%; *P* = 0.03), gastrointestinal diseases (11.2 versus 6.3%; *P* = 0.008), non-diabetes endocrine disorders (9.6 versus 5.3%; *P* = 0.01) and traumatic brain injury (7.1 versus 3.3%; *P* = 0.01). Comorbidities are shown in Table 2.

**Table 2:** Comorbidities for PVI and BTI in PNP and NNP patients

	Overall PNP	PNP PVI	PNP BTI	*P*	Overall NNP	NNP PVI	NNP BTI	*P*
*n*	200	174	26		1100	778	322	
Any pre-existing comorbidity *n* (%)								
Hypertension	71 (35.5)	61 (35.1)	10 (38.5)	0.8	175 (15.9)	115 (14.8)	60 (18.6)	0.1
Type 2 diabetes	48 (24)	40 (22.9)	8 (30.8)	0.5	51 (4.6)	35 (4.5)	16 (4.9)	0.8
Dyslipidaemia	38 (19)	31 (17.8)	7 (26.9)	0.27	141 (12.8)	91 (11.7)	50 (15.5)	0.09
Depression/anxiety	34 (17)	25 (14.4)	9 (34.6)	**0**.**02**	266 (24.2)	171 (21.9)	95 (29.5)	**0**.**01**
Lung disease	30 (15)	26 (14.9)	4 (15.4)	1	188 (17.1)	117 (15)	71 (22)	**0**.**006**
Autoimmune disease	25 (12.5)	19 (10.9)	6 (23.1)	0.1	134 (12.2)	86 (11.1)	48 (14.9)	0.08
Cancer	22 (11)	18 (10.3)	4 (15.4)	0.5	61 (5.5)	42 (5.4)	19 (5.9)	0.7
Other endocrine disorders	20 (10)	18 (10.3)	2 (7.7)	1	72 (6.5)	41 (5.3)	31 (9.6)	**0**.**01**
Gastrointestinal disease	18 (9)	14 (8)	4 (15.4)	0.3	85 (7.7)	49 (6.3)	36 (11.2)	**0**.**008**
Headache	13 (6.5)	10 (5.7)	3 (11.5)	0.3	150 (13.6)	94 (12.1)	56 (17.4)	**0**.**03**
Chronic kidney disease	8 (4)	7 (4)	1 (3.8)	1	14 (1.3)	8 (1)	6 (1.9)	0.3
Insomnia	8 (4)	7 (4)	1 (3.8)	1	82 (7.5)	55 (7.1)	27 (8.4)	0.5
Cardiovascular disease	8 (4)	6 (3.4)	2 (7.7)	0.3	33 (3)	19 (2.4)	14 (4.3)	0.1
Peripheral vascular disease	5 (2.5)	4 (2.3)	1 (3.8)	0.5	8 (0.7)	5 (0.6)	3 (0.9)	0.7
Dysautonomia	5 (2.5)	4 (2.3)	1 (3.8)	0.5	18 (1.6)	9 (2.8)	9 (1.2)	**0**.**06**
Cerebrovascular disease	5 (2.5)	5 (2.9)	0 (0)	1	9 (0.8)	5 (0.6)	4 (1.2)	0.3
Neuropsychiatric disease	3 (1.5)	2 (1.1)	1 (3.8)	0.3	53 (4.8)	31 (3.9)	22 (6.8)	0.06
Traumatic brain injury	3 (1.5)	2 (1.1)	1 (3.8)	0.3	49 (4.5)	26 (3.3)	23 (7.1)	**0**.**01**
Neuromuscular disease	1 (0.5)	1 (0.6)	0 (0)	0.1	3 (0.3)	1 (0.1)	2 (0.6)	0.2
Organ transplant	1 (0.5)	0 (0)	1 (3.8)	0.1	1 (0.1)	0 (0)	1 (0.3)	0.3
Other	57 (28.5)	49 (28.2)	8 (30.8)	0.8	300 (27.3)	198 (25.4)	102 (31.7)	**0**.**04**

*P*-values that are significant *P* < 0.05 are highlighted in bold.

### Frequency of neurological symptoms and signs attributed to COVID-19

PNP patients had their clinic visits an average of 9.6 months after symptom onset, with no statistically significant difference between PNP PVI and PNP BTI. NNP patients were seen in our clinic an average of 10 months after symptom onset, with NNP BTI being evaluated earlier than NNP PVI patients (8.9 versus 10.5 months; *P* < 0.0001). In terms of subjective impression of recovery compared to their pre-COVID-19 baseline, PNP patients had a mean recovery of 55.5% from their baseline, with no significant difference between PNP PVI and PNP BTI. NNP patients had a mean recovery of 57.7%, also with no significant differences between NNP PVI and NNP BTI. The median number of neurologic symptoms attributed to PASC was 5 for PNP and 6 for NNP groups. Also, 95% of PNP patients and 90.4% of NNP patients reported more than four neurological symptoms, with no significant difference between PNP PVI and PNP BTI, or between NNP PVI and NNP BTI.

The 10 most common neurological symptoms reported by PNP patients include brain fog (86.5%), headache (56.5%), numbness/tingling (56.5%), dizziness (55.5%), myalgia (53%), pain other than chest (46.5%), dysgeusia (43.5%), anosmia (42%), tinnitus (33.5%) and blurred vision (30%). Of these symptoms, PNP BTI more frequently reported anosmia compared to PNP PVI patients (69.2 versus 37.9%; *P* = 0.003). Seizures, movement disorders, ischaemic stroke, encephalitis, focal sensory deficits and meningitis were rare in both PNP groups. None of the PNP patients presented with focal motor deficits, haemorrhagic stroke or polyradiculitis. For NNP patients, the 10 most common neurological symptoms were brain fog (83.9%), headache (70.9%), dizziness (53.8%), myalgia (53.2%), anosmia (51.5%), dysgeusia (48.6%), pain other than chest (43.7%), numbness/tingling (41.5%), tinnitus (33.2%) and blurred vision (31.5%). NNP PVI more often than NNP BTI patients reported anosmia (56.6 versus 39.1%; *P* < 0.0001) and dysgeusia (53.3 versus 37.3%; *P* < 0.0001). However, NNP BTI more frequently reported dizziness compared to NNP PVI patients (61.5 versus 50.6%; *P* = 0.001). Seizures, movement disorders, ischaemic stroke, encephalitis, focal motor deficits, focal sensory deficits and haemorrhagic stroke were rare in both NNP groups. None of the NNP patients presented with meningitis or polyradiculitis.

The most common non-neurological symptoms for PNP patients were fatigue (86%), shortness of breath (70%), depression/anxiety (68.5%), insomnia (61.5%), chest pain (40%), dysautonomia (33.5%) and gastrointestinal symptoms (23%). There was no statistically significant difference in prevalence of non-neurological symptoms between PNP PVI and PNP BTI patients. For NNP patients, fatigue (87.5%), depression/anxiety (69.4%), insomnia (57%), shortness of breath (46.5%), dysautonomia (36.2%), chest pain (30.4%) and gastrointestinal (GI) symptoms (27.2%) were the most common non-neurological symptoms. Of these, NNP BTI more frequently than NNP PVI patients reported dysautonomia (40.1 versus 34.6%; *P* = 0.002) and GI symptoms (32.3 versus 25.1%; *P* = 0.01). We performed a complete neurologic physical exam on the 709 patients who came to the clinic in-person and a limited exam for the 591 patients who were seen via televisits. Among the NNP patients, BTI was more likely to show attention deficit on neurologic exam compared to PVI (15.3 versus 9.8%, *P* = 0.02). All other PNP and NNP groups had no statistically significant differences in physical exam findings. Complete neurological signs and symptoms are shown in Table 3.

**Table 3:** Neurologic symptoms and signs attributed to long COVID for PVI and BTI in PNP and NNP patients

	Overall PNP	PNP PVI	PNP BTI	*P*	Overall NNP	NNP PVI	NNP BTI	*P*
*n*	200	174	26		1100	778	322	
Months since symptom onset [mean (1 SD)]	9.6 (6.3)	9.7 (6.3)	8.6 (6.2)	0.32	10 (6.7)	10.5 (6.5)	8.9 (6.9)	**<0**.**0001**
Recovery from pre-COVID baseline [mean % (1 SD)]	55.6 (25.3)	56.3 (24.4)	50.2 (30.2)	0.41	57.7 (24.5)	58.1 (24.1)	56.7 (25.4)	0.64
Neurologic manifestations or symptoms attributed to PASC [median (IQR)] *n* (%)	5 (3–7)	5 (3–7)	6.5 (3–7.3)	0.21	5 (3–7)	5 (3–7)	5 (3–7)	0.45
≥4	138 (69)	121(69.5)	17 (65.4)	0.7	797 (72.4)	570 (73.3)	227 (70.5)	0.35
Brain fog	173 (86.5)	151 (86.8)	22 (84.6)	0.76	923 (83.9)	643 (82.6)	280 (87)	0.09
Headache	113 (56.5)	98 (56.3)	15 (57.7)	0.9	780 (70.9)	548 (70.4)	232 (72)	0.61
Numbness/tingling	113 (56.5)	94(54)	19 (73.1)	0.07	456 (41.5)	328 (42.2)	128 (39.8)	0.50
Dizziness	111 (55.5)	97(55.7)	14(53.8)	0.86	592 (53.8)	394 (50.6)	198 (61.5)	**0**.**001**
Myalgia	106 (53)	91 (52.3)	15 (57.7)	0.61	585 (53.2)	416 (53.5)	169 (52.5)	0.79
Pain other than chest	93 (46.5)	81 (46.6)	12(46.2)	0.97	481 (43.7)	336 (43.2)	145 (45)	0.59
Dysgeusia	87 (43.5)	72(41.4)	15(57.7)	0.12	535 (48.6)	415 (53.3)	120 (37.3)	**<0**.**0001**
Anosmia	84 (42)	66(37.9)	18 (69.2)	**0**.**003**	566 (51.5)	440 (56.6)	126 (39.1)	**<0**.**0001**
Tinnitus	67 (33.5)	59 (33.9)	8 (30.8)	0.75	365 (33.2)	246 (31.6)	119 (37)	0.09
Blurred vision	60 (30)	51(29.3)	9(34.6)	0.58	346 (31.5)	239 (30.7)	107 (33.2)	0.43
Ischaemic stroke	5 (2.5)	4 (2.3)	1 (3.8)	0.51	15 (1.4)	10 (1.3)	5 (1.6)	0.78
Seizure	4 (2)	4 (2.3)	0 (0)	1	21 (1.9)	16 (2.1)	5 (1.6)	0.8
Meningitis	2 (1)	2 (1.1)	0 (0)	1	0 (0)	0 (0)	0 (0)	1
Movement disorder	2 (1)	1 (0.6)	1 (3.8)	0.24	6 (0.5)	5 (0.6)	1 (0.3)	0.68
Encephalitis	1 (0.5)	1 (0.6)	0 (0)	1	1 (0.1)	1 (0.1)	0 (0)	1
Focal sensory deficit	1 (0.5)	1 (0.6)	0 (0)	1	3 (0.3)	2 (0.3)	1 (0.3)	1
Focal motor deficit	0 (0)	0 (0)	0 (0)	1	2 (0.2)	1 (0.1)	1 (0.3)	0.5
Haemorrhagic stroke	0 (0)	0 (0)	0 (0)	1	1 (0.1)	1 (0.1)	0 (0)	1
Polyradiculitis	0 (0)	0 (0)	0 (0)	1	0 (0)	0 (0)	0 (0)	1
Other symptoms *n* (%)								
Fatigue	172 (86)	151 (86.8)	21 (80.8)	0.38	963 (87.5)	675 (86.8)	288 (89.4)	0.23
Shortness of breath	140 (70)	123 (70.7)	17 (65.4)	0.65	511 (46.5)	358 (46)	153 (47.5)	0.1
Depression/anxiety	137 (68.5)	120 (69)	17 (65.4)	0.71	763 (69.4)	544 (69.9)	219 (68)	0.57
Insomnia	123 (61.5)	108 (62.1)	15 (57.7)	0.67	627 (57)	431 (55.4)	196 (60.9)	0.11
Chest pain	80 (40)	69 (39.7)	11 (42.3)	0.8	334 (30.4)	232 (29.8)	102 (31.7)	0.57
Dysautonomia	67 (33.5)	58 (33.3)	9 (34.6)	0.90	398 (36.2)	269 (34.6)	129 (40.1)	**0**.**002**
GI symptoms	46 (23)	38 (21.8)	8 (30.8)	0.31	299 (27.2)	195 (25.1)	104 (32.3)	**0**.**02**
Sign *n* tested/total (%)	190/200 (95)	164/174 (94.3)	26/26 (100)	0.37	1019/1100 (92.6)	725/778 (93.2)	294/322 (91.3)	0.31
Abnormal exam^a^	110 (55)	94 (57.3)	16 (51.5)	0.47	406 (39.8)	279 (38.5)	127 (43.5)	0.18
Memory deficit	68 (34)	58 (35.4)	10 (38.5)	0.61	263 (25.8)	179 (24.7)	84 (28.6)	0.21
Attention deficit	33 (16.5)	30 (18.3)	3 (11.5)	0.58	116 (11.4)	71 (9.8)	45 (15.3)	**0**.**02**
Sensory dysfunction	32 (16.8)	28 (17.1)	4 (15.4)	1	74 (7.3)	48 (6.6)	26 (8.8)	0.23
Gait dysfunction	31 (16.3)	26 (15.9)	5 (19.2)	0.57	47 (4.6)	29 (4)	18 (6.1)	0.18
Motor dysfunction	22 (11.6)	20 (12.2)	2 (7.7)	0.74	29 (2.8)	23 (3.2)	6 (2)	0.41
Cranial nerve dysf.	7 (3.7)	7 (4.3)	0 (0)	0.6	22 (2.2)	20 (2.8)	2 (0.7)	0.05
Cerebellar dysf.	4 (2.1)	4 (2.3)	0 (0)	1	6 (0.6)	4 (0.6)	2 (0.7)	1
Movement disorder	4 (2.1)	3 (1.8)	1 (3.8)	1	15 (1.5)	11 (1.5)	4 (1.4)	1

^a^Calculated based on *n* tested. Dysf, dysfunction. *P*-values that are significant *P* < 0.05 are highlighted in bold.

### Quality-of-life measures and standardized cognitive tests

**Figure 1:**
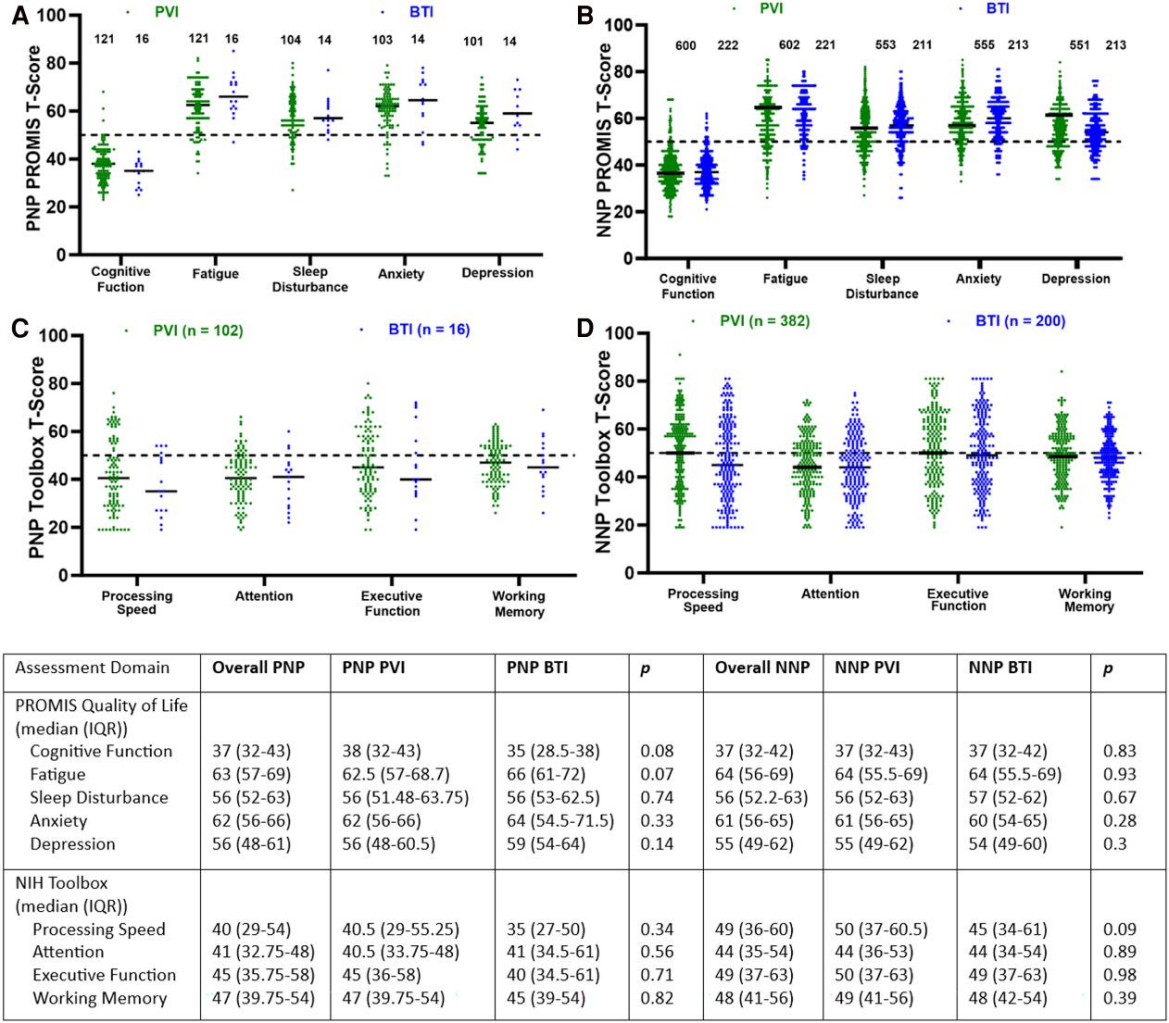
**Quality-of-life and cognitive results of PNP and NNP in PVI and BTI patients.** PROMIS *T*-score for PNP PVI and BTI (**A**) and NNP PVI and BTI (**B**). NIH toolbox *T*-score for PNP PVI and BTI (**C**) and NNP PVI and BTI (**D**). PROMIS and NIH toolbox scores were compared between groups using the Mann–Whitney U-test and two-sided *P* ≤ 0.05 was considered statistically significant. There were no statistically significant differences in the quality-of-life assessments between the PNP PVI and PNP BTI patients or between the NNP PVI and NNP BTI patients. Similarly, there was no statistically significant difference in the NIH toolbox scores between the PNP PVI and PNP BTI patients, nor the NNP PVI and NNP BTI patients.

The results for the PROMIS quality-of-life and NIH Toolbox cognitive scores are reported in Fig. 1. Within the PNP group, there was no statistically significant difference in the quality-of-life measures between those who had a PVI and a BTI. Similarly, there was no statistically significant difference in the quality-of-life measures in the NNP PVI and NNP BTI patients. The median *T*-scores for each of these groups indicated moderate impairment in quality-of-life, which was significantly worse than a US normative population (Table 4).

**Table 4:** Quality-of-life and cognitive results of PNP and NNP patients compared to a US normative population

Assessment domain	Overall PNP	*P*, norm^a^	Overall NNP	*P*, norm^a^
PROMIS quality-of-life [median (IQR)]				
Cognitive function	37 (32, 43)	**<0.0001**	37 (32, 42)	**<0.0001**
Fatigue	63 (57, 69)	**<0.0001**	64 (56, 69)	**<0.0001**
Sleep disturbance	56 (52, 63.5)	**<0.0001**	56 (52, 63)	**<0.0001**
Anxiety	62 (56, 66)	**<0.0001**	61 (56, 65)	**<0.0001**
Depression	56 (48, 60.3)	**<0.0001**	55 (49, 62)	**<0.0001**
NIH toolbox [median (IQR)]				
Processing speed	40 (29, 54)	**<0.0001**	49 (36, 61)	**0.02**
Attention	40.5 (32.8, 48)	**<0.0001**	44 (36, 53)	**<0.0001**
Executive function	45 (35.8, 58)	**0.01**	50 (37, 63)	0.99
Working memory	47 (39.8, 54)	**<0.0001**	48 (41, 56)	**0.0009**

^a^ *P-*values that are significant *P* < 0.05 when comparing PNP and NNP patients to a normative US population are highlighted in bold.

There were 119 PNP patients who received the NIH Toolbox assessment, among whom 102 were PVI patients and 16 were BTI patients. Furthermore, there were 582 NNP patients who received the NIH Toolbox assessment, with 382 PVI and 200 BTI patients. There was no statistically significant difference in the objective measures of cognitive function between the PNP PVI and PNP BTI patients. Similarly, there was no statistically significant difference in the NIH Toolbox scores between the NNP PVI and NNP BTI patients. However, PNP patients performed worse on NIH Toolbox tests of processing speed, attention, executive function and working memory than a US normative population whereas NNP patients had lower results in processing, speed, attention and working memory (Table 4).

### Assessment of subjective recovery to pre-COVID-19 baseline

**Figure 2:**
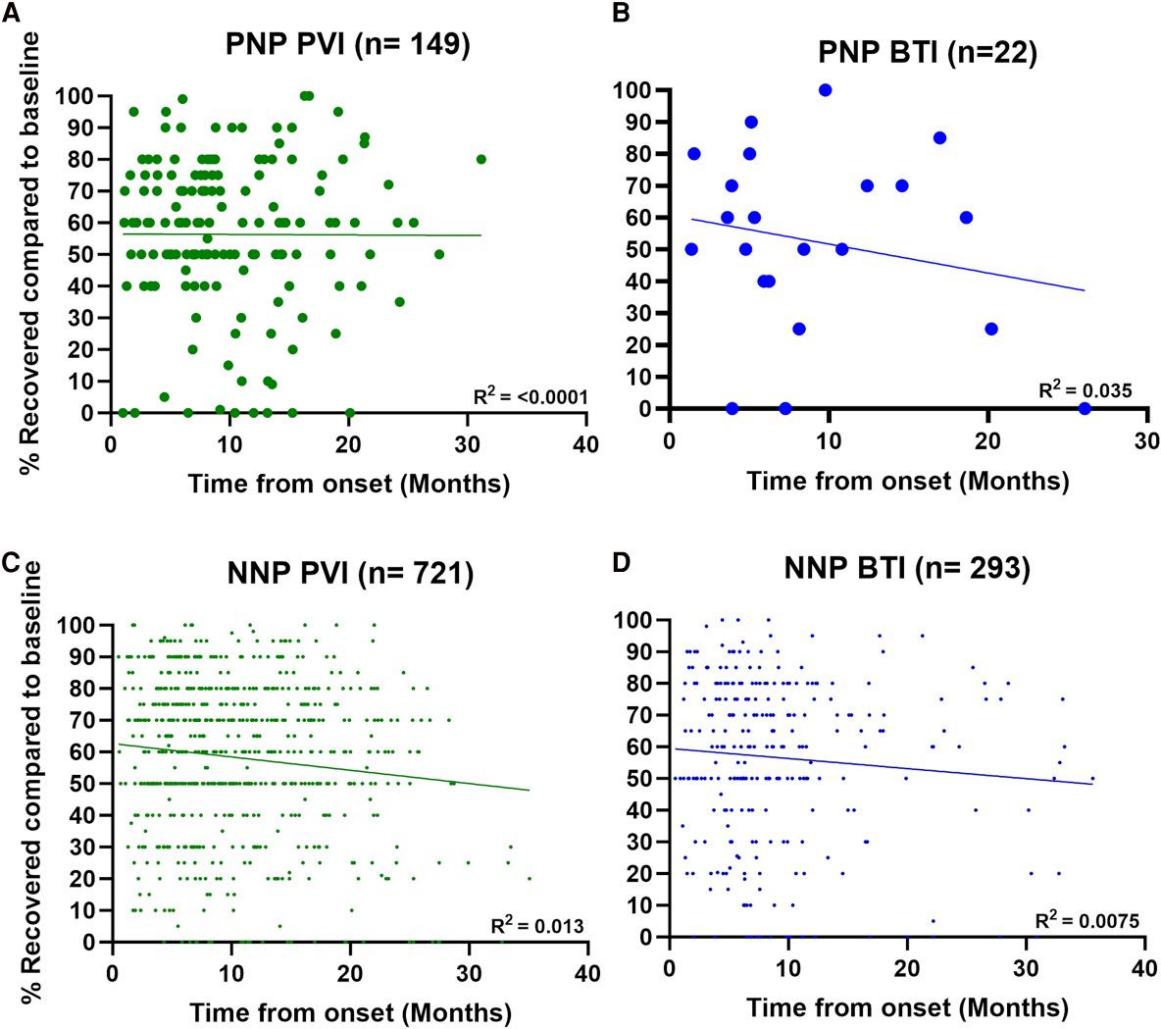
**Recovery to pre-COVID baseline in PNP and NNP for PVI and BTI patients.** Subjective impression of recovery compared to pre-COVID-19 baseline for PNP PVI (**A**) and PNP BTI (**B**), as well as NNP PVI (**C**) and NNP BTI (**D**). The patients were asked to grade their recovery at the time of their visit, assuming a pre-COVID-19 baseline of 100%. Each person is represented by a single time point. We determined Spearman’s rank correlation coefficients (expressed as *R*^2^ values) and considered correlations statistically significant if two-sided *P* ≤ 0.05. In each instance, correlations demonstrated no significant relationship between time from onset and per cent recovery in both PNP and NNP.

We analysed the subjective impression of per cent recovery compared to pre-COVID-19 baseline in PNP and NNP patients at the time of the visit for both PVI and BTI. Time from symptom onset was not associated with the subjective impression of recovery in either group (Fig. 2).

### Multiple correspondence analysis

**Figure 3:**
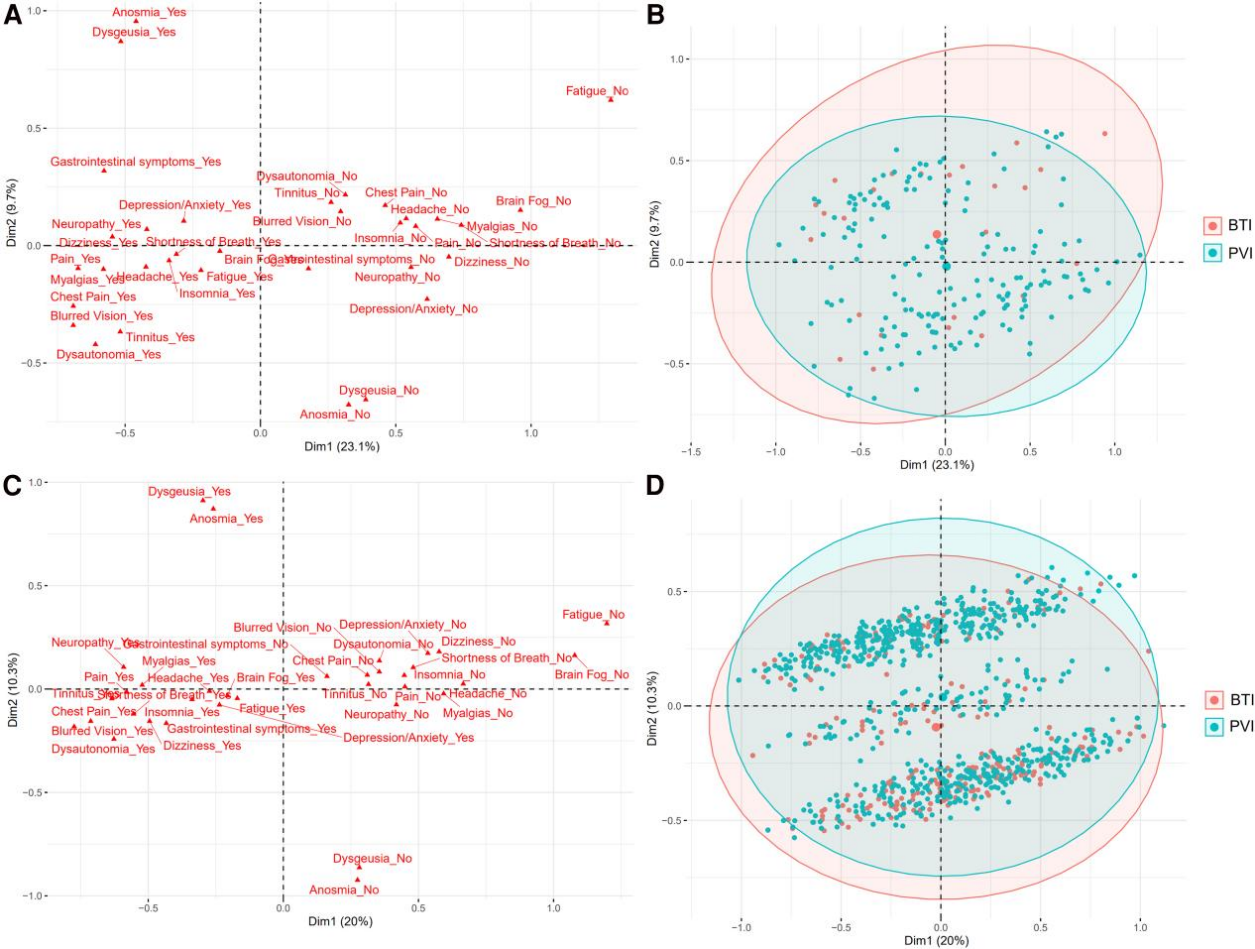
**Multiple correspondence analysis.** (**A**) PNP cohort symptom point cloud. (**B**) PNP cohort patient point cloud with vaccination group concentration ellipses. Larger sized points represent the centre of each vaccination groups’ distribution. (**C**) NNP cohort symptom point cloud. (**D**) NNP cohort patient point cloud with vaccination group concentration ellipses. Larger sized points represent the centre of each vaccination groups’ distribution. Increasing distance between the origin and a given symptom category indicates a greater contribution of that category to the pole of the corresponding dimension. Symptom categories with similar profiles of patients are grouped together. For both PNP and NNP patients, 1D globally separates ‘yes’ symptom categories (*left* part of the graph, smaller 1D values) from ‘no’ symptom categories (*right* part of the graph, larger 1D values). For both PNP and NNP patients, the presence or absence of dysgeusia and anosmia are the predominant contributors to 2D. This results in a visually stratified patient cloud (particularly for the NNP cohort) along 2D depending on the presence or absence of both dysgeusia and anosmia. 1D values did not differ significantly between vaccination groups among either PNP or NNP cohorts. However, 2D differed significantly between vaccination group. The PNP BTI group had significantly larger 2D values than the PVI group (Welch two sample *t*-test; *t* = 2.13, df = 30.46; *P* = 0.04) suggesting more frequent composite of anosmia/dysgeusia. In contrast, the NNP BTI group had significantly smaller 2D values than the PVI group (Welch two sample *t*-test; *t* = −6.39, df = 622.68; *P* < 0.001) suggesting less frequent composite of anosmia/dysgeusia.

Seventeen symptoms were reported as present in 20% or more of patients and were included in the MCAs. For the PNP cohort, 1D explained 23.1% of the variance while 2D explained 9.7% of the variance; each of the remaining 15D explained 7.8% or less of the variance. Five symptoms had correlation coefficient squared values (*R*^2^) with PNP 1D that exceeded 0.25: pain (0.39), dizziness (0.38), myalgias (0.38), chest pain (0.32) and fatigue (0.28) with all other symptoms having *R*^2^ values between 0.10 and 0.23. For PNP 2D, only two symptoms had *R*^2^ values exceeding 0.25: anosmia (0.65) and dysgeusia (0.57) with all other symptoms having *R*^2^ values below 0.09. For the NNP cohort, 1D explained 20.0% of the variance while 2D explained 10.3% of the variance; each of the remaining 15D explained <8.0% of the variance. Six symptoms had *R*^2^ values with NNP 1D that exceeded 0.25: myalgias (0.31), dizziness (0.29), blurred vision (0.27), shortness of breath (0.27), pain (0.26) and neuropathy (0.25) with all other symptoms having *R*^2^ values between 0.07 and 0.22. For NNP 2D, only two symptoms had *R*^2^ values exceeding 0.25: anosmia (0.80) and dysgeusia (0.79) with all other symptoms having *R*^2^ values below 0.03. Figure 3A–D shows the patient and symptom point clouds for the PNP and NNP cohorts, and the figure caption provides the interpretation of the MCA graphs. For both PNP and NNP cohorts, the PVI and BTI groups did not significantly differ in 1D, suggesting similar profiles for the symptoms composing 1D. For the PNP group, BTI patients had significantly higher values for 2D than PVI patients (mean: 0.137 ± 0.358 versus −0.020 ± 0.300; *P* = 0.04), suggesting that PNP BTI patients more frequently reported the composite of anosmia/dysgeusia. In contrast, for the NNP group, BTI patients had significantly lower values for 2D than PVI patients (mean: −0.093 ± 0.306 versus 0.038 ± 0.319; *P* < 0.001), suggesting that BTI NNP patients less frequently reported the composite of anosmia/dysgeusia.

## Discussion

Our previous work has demonstrated the importance in evaluating PNP and NNP patients separately.^5^ However, it is also critical to develop a better understanding of Neuro-PASC in the PVI and BTI settings. This present study addresses a key knowledge gap regarding the impact of vaccination on Neuro-PASC symptoms in both post-hospitalization and non-hospitalized patients.

Although we started to see patients with BTI at the beginning of 2021 soon after vaccines became available in the US, there was no difference in demographics compared to PVI patients evaluated since the opening of the clinic in May 2020 in both PNP and NNP groups. However, we observed differences in the patients’ comorbidities, with a higher frequency of patients with prior history of depression/anxiety in both PNP and NNP BTI groups compared to their respective PVI groups. Additionally, the NNP BTI group had a greater prevalence of headache, endocrine disorders, lung and gastrointestinal diseases, traumatic brain injury and other comorbid conditions compared to the PVI group.

These results suggest that long COVID may occur in patients with a higher burden of comorbidities in the BTI setting, especially in the NNP group. In particular, the higher prevalence of depression/anxiety prior to COVID-19 in the BTI group suggest that pre-existing mental health issues contribute to the vulnerability of developing long COVID in those patients. These findings are consistent with a prospective study where psychological distress was associated with a higher risk of developing post-COVID conditions and a lower likelihood of full recovery.^27^ One possible explanation for the difference in psychiatric comorbidities between PVI and BTI groups, is that BTI patients had their initial infection later on than PVI patients, after they had been exposed to the stress of the pandemic for longer periods of time than the PVI group, consistent with dramatic increases in overall global prevalence of depression and anxiety as the pandemic continued.^28^

Conversely, we found no significant differences for most of the symptoms and signs of long COVID between the PVI and BTI groups. After an average of 10 months from symptom onset, patients felt <60% recovered compared to their pre-COVID baseline and had multiple neurologic symptoms or manifestations attributed to PASC. Among PNP patients, all but one of the symptoms (anosmia) had a similar prevalence in the PVI and BTI groups. The NNP patients had very similar presentations of long COVID as well; the only differences were a higher frequency of dizziness and attention deficit as well as a decreased frequency of anosmia and dysgeusia in the BTI group.

This was also demonstrated in the MCA, which showed that BTI patients in the NNP group less frequently had a combination of anosmia and dysgeusia than those who had PVI. Interestingly, the MCA showed the converse in the PNP group, BTI patients more frequently reported anosmia and dysgeusia than PVI patients. Our study is not designed to identify the reason for this divergent finding of anosmia/dysgeusia between PNP and NNP patients in terms of BTI versus PVI. A possibility is that many NNP PVI patients were affected by the initial strain of SARS-CoV-2, which more frequently caused disorders of smell and taste than subsequent variants affecting NNP BTI patients (strain effect).^29,30^ However, anosmia has also been associated with less severe COVID-19 presentation.^31,32^ This may explain why PNP BTI more frequently had anosmia than PNP PVI patients, since they had some protection conferred by the vaccine, which had been shown to decrease the severity of the initial infection in hospitalized individuals (severity effect).^33^ Other than anosmia/dysgeusia however, the MCA demonstrated that the symptom profiles were similar between the PVI and BTI groups in both NNP and PNP patients.

We previously showed the subjective alterations in the quality-of-life of PNP and NNP patients across domains of cognition, fatigue, sleep disturbance, anxiety and depression, as well as the distinct patterns of objective measures of cognition between the two groups.^5^ The present study confirms those findings but showed no differences in quality-of-life or cognitive dysfunction between individuals with PVI and BTI in both PNP and NNP groups. These results indicate that the core subjective and objective manifestations of Neuro-PASC are similar in both the setting of PVI and BTI. We also demonstrated previously that time from symptom onset was not associated with subjective recovery compared to baseline in PNP and NNP patients.^5^ We confirm here that this is also the case for individuals with PVI and BTI in both groups.

Taken together, these results indicate that, once PNP or NNP patients develop Neuro-PASC, whether they contracted SARS-CoV-2 infection prior to, or after SARS-CoV-2 vaccination makes little difference in their clinical presentation, subjective alteration of quality-of-life or objective cognitive dysfunction. However, our data suggest that it may take a higher burden of comorbidities to develop Neuro-PASC as a BTI, especially in NNP patients. Furthermore, the higher prevalence of depression/anxiety prior to COVID-19 in both PNP and NNP patients who developed Neuro-PASC after BTI compared to PVI highlights a potentially preventable psychiatric vulnerability.

As of July 1, 2024, the Center for Disease Control and Prevention reported that 82% of people in the USA have received at least one dose of COVID-19 vaccine. Although vaccines have markedly decreased the incidence of severe COVID-19, hospitalizations and deaths,^34^ they incompletely protect against new SARS-CoV-2 infection.^35^ While long COVID was initially defined prior to the availability of COVID-19 vaccines, most new infections now occur in vaccinated individuals. Our study is novel, as it is the first to compare the clinical characteristics, quality-of-life and cognitive function of Neuro-PASC patients who contracted SARS-CoV-2 as PVI or BTI. Our results provide important additional knowledge compared to previous studies that had mainly focused on variation in incidence of long COVID in patients infected after vaccination,^8-12^ or included solely patients previously hospitalized for COVID-19 pneumonia.^36^

Our study has limitations. Our definition of long COVID differed from the CDC and WHO definitions regarding duration of symptoms (6 weeks in our study, compared to 4 weeks and 12 weeks for CDC and WHO, respectively). However, our definition had already been established prior to that of either of these organizations and >90% of our patients fit the WHO definition. The patients included in this study were individuals who chose to seek care at Northwestern’s Neuro-COVID-19 clinic. This is similar to other studies researching diseases in healthcare settings or via online questionnaires, where patients seeking care self-select based on a multitude of factors, including geography, technological access and socio-economic status.

We did not require physician referral to increase access to care for patients across the entire USA. As such, our study population coming from 37 states is representative of those who seek care in post-COVID clinics in the USA as a whole. Though study visits were designed to be as uniform as possible between in-person and telehealth visits, telehealth visits limit the extent of the physical exam that can be performed. Cognitive performance of each patient was assessed with both the NIH Toolbox and PROMIS score. Both measures have been validated extensively for neurological research, including using normative population data for comparison, which was necessary as we were unable to test our own control groups due to limitations on human subjects’ research during the pandemic. Our statistical analyses did not adjust for multiple covariates. This is due to the exploratory nature of this first-of-its-kind study aiming at guiding further investigations, since those adjustments may increase the type II error for those associations that are not null.^37,38^

There were more NNP compared to PNP patients, consistent with the evolution of the pandemic and the observation that most SARS-CoV-2 infections do not require hospitalization, providing a larger power to determine differences between PVI and BTI in the NNP patient group. Moreover, our study coincided with the widespread introduction of vaccination; due to the protective effect of vaccination against infection, PVI was more common than BTI in our cohort. This likely explains the inequal number of patients in the PVI and BTI groups, but we prioritized the decision to study consecutive patients visiting the clinic.

Only patients with PVI were infected by the initial strain of SARS-CoV-2 in 2020 while both patients with PVI and BTI could have been infected with the Delta variant in the Spring of 2021 or the Omicron variants in late 2021 and onwards. This is based on epochs only, as there was no method to retrospectively confirm the exact viral strain for each patient.

## Conclusion

Due to the combination of vaccines and strain evolution, COVID-19 is currently a mild respiratory condition that rarely requires hospitalization. Although PASC has remained a multi-organ disease, it now predominantly affects the nervous system.^4,5^ Indeed, the cognitive impairment of PASC was found to be of the same magnitude as alcohol intoxication at the UK/USA driving limit (80 mg/100 mL blood or 35 mcg/100 mL breath) or 10 years of cognitive aging.^39^ While vaccination decreases the severity of acute COVID-19 and the rate of hospitalization and death, the sobering conclusion of our study is that vaccination prior to infection did not alter the subsequent neurologic manifestations of long COVID in our clinic population. Patients developing Neuro-PASC after BTI have a higher burden of comorbidities than those with PVI, highlighting different risk factors warranting targeted management. Further longitudinal studies are warranted to determine the long-term outcome of neuro-PASC patients with PVI and BTI.^40-43^

## Data Availability

Data will be deposited in the COVID-19 NeuroData Bank after publication.
